# Moral dilemmas of animal production systems

**DOI:** 10.1093/af/vfz051

**Published:** 2020-01-10

**Authors:** Bart Gremmen

**Affiliations:** Department of Philosophy, Wageningen University, Wageningen, The Netherlands

**Keywords:** animal production systems, animal welfare, care ethics, emergent ethics, moral lock-in

**Implications**

- An ethics of animal production “systems” consists of a Moral Operating System.
- A Moral Operating System consists of an “internal” professional “care” ethics, an “external animal” ethics, and an “emergent” ethics in life sciences enabling change by responsible innovation.
- It is important to broaden the existing ethical frameworks on agriculture to new scientific methods and technologies.
- A Moral Operating System will help scientists, stakeholders, and policymakers to understand, evaluate, and monitor the integration of ethical aspects of agricultural systems.

## Introduction

Globally, the number of animals used by humans has grown to an all-time high. Hundreds of millions of animals play an important role in different activities as pets, in sports, as hobby and companion animals, but they are also used in clinical trials and tests, especially in agriculture (Eijsackers and Scholten, 2011). However, many societies are changing dramatically in relation to societal ideas about animal production (Ankersmit, 2010), especially about genetic modification and the use of animals (Bruijnis et al., 2015). To mention only a few trends that will reduce animal numbers in the long run: a ban on wild circus animals, a restriction on keeping certain species as pets, and a reduction in dairy cows and pigs. Besides their role in society, animals are also found in nature areas, where humans are causing the sixth global mass extinction of wild animals.

**Figure 1.**
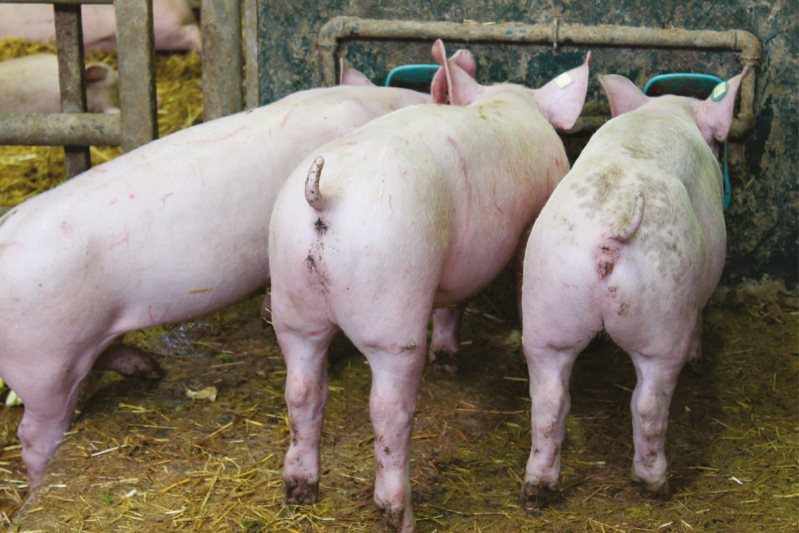
Pigs with docked tails.

Hereafter, I will focus on the moral dilemmas of animal production systems. Examples are tail docking of piglets (Figure 1) and killing day-old male chicks.

These moral dilemmas arise when societal values clash with the principles of an animal production system because of unintended consequences and risks. To tackle these moral dilemmas as an ethicist, it is necessary not only to be part of a life sciences trajectory of responsible innovation but also to strengthen ethical reflection along the agricultural production chains and among the involved stakeholders.

## Ethics and Animals

Clashes of different ethical approaches may be observed in societal debates about animals (Thompson, 1998), and I will illustrate this by using the case of Johannes, a humpback whale.

On 12 December 2012, Johannes beached on the shoreline of De Razende Bol, a small uninhabited island between the island of Texel and the city of Den Helder in The Netherlands.

**Figure 2.**
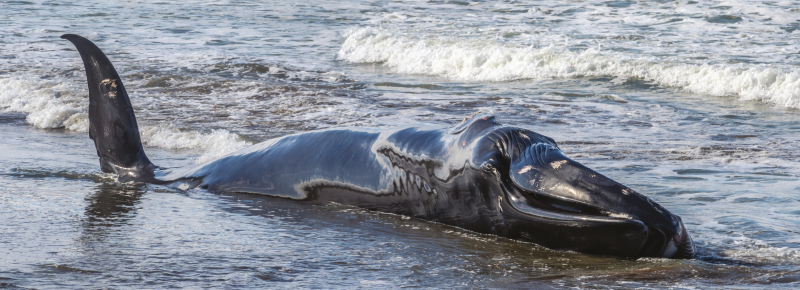
Wounded humpback whale grounded in the coast near Sopelana, Basque Country.

The whale was stuck on the beach and could not return to the water on its own (Figure 2). Not so long ago, humans living nearby would have killed the animal immediately, and its remains would have been used for all kinds of purposes. In our modern times, we try to save such animals’ lives. Over the course of just a few days, Johannes became a national symbol for helping a wild animal in need. Political parties, civil servants, scientists, and members of societal organizations were engaged not only in debates but also in rescue and euthanasia attempts. All these attempts failed, and the whale eventually died. In the ensuing debate, ecologists and nature conservationists still argued against killing dying wild animals in distress, whereas the majority of the other participants in the debate argued for a humane death for these animals.

At first sight, it seems that humans do not need to be involved at all when wild animals die: wild animals are wild precisely because they “take care of” themselves in areas where they are outside human control. In such situations, wild animals die for several different reasons: hunger, thirst, disease, predators, and also as a consequence of old age. Humans are often unaware of the fact that, out of their sight, animals could be dying. However, sometimes people are confronted with dying wild animals, like the beached humpback whale in 2012. What is our moral reference for killing wild animals? It seems that, in order to answer these questions, we can rely on animal ethics: the moral framework for the killing of domesticated animals. According to the law in most European countries, humans are obliged to help an animal in distress. From this ethical perspective, the first duty of humans is to save or help individual wild animals in situations where humans are present. When all help fails, our second duty is, if possible, to kill these animals in a humane way. The example of the humpback whale seems to fit into this scheme because it was an individual animal surrounded by humans. However, from the perspective of eco-ethics, wild animals are part of ecosystems. Therefore, the focus is on groups and species rather than on individual animals. In general, this ethical framework advocates respect for the wildness of animals. In the case of dying wild animals, like the humpback whale, the eco-ethics ethical framework advises a hands-off strategy. This seems to lead to a stalemate between two rival ethical frameworks, thus leaving nature management caught between two sets of norms governing animals and nature.

If we see some ethological distance in the dualism between “wild” and “tame”, all kinds of intermediary shades appear. Also, the number of situations in which humans have to decide to kill wild animals increases considerably. The humpback whale is an example of an individual wild animal in distress. We may consider this situation as bad luck and exceptional. But what about lost or abandoned baby seals on the shores of The Netherlands, Germany, and Denmark? When we locate these animals, do we help them by bringing them to a shelter? Do we have to kill them on the spot or leave them alone to die? Other examples are weak or dying animals in nature parks like the Oostvaardersplassen in The Netherlands (Gremmen, 2016) and exotic animals that are destroying the biodiversity of an area. In earlier research (Gremmen, 2016), we argued that the relation between animal ethics and eco-ethics in these cases is not a dichotomy but a continuum.

Because animal ethics is about individual animals in hands-on situations, and eco-ethics is about groups of animals and species in hands-off situations; groups of animals in agricultural hands-on situations do not belong to either ethics. What is a suitable ethics of animals in agricultural production systems?

## Ethics of Agricultural Production Systems

**Figure 3.**
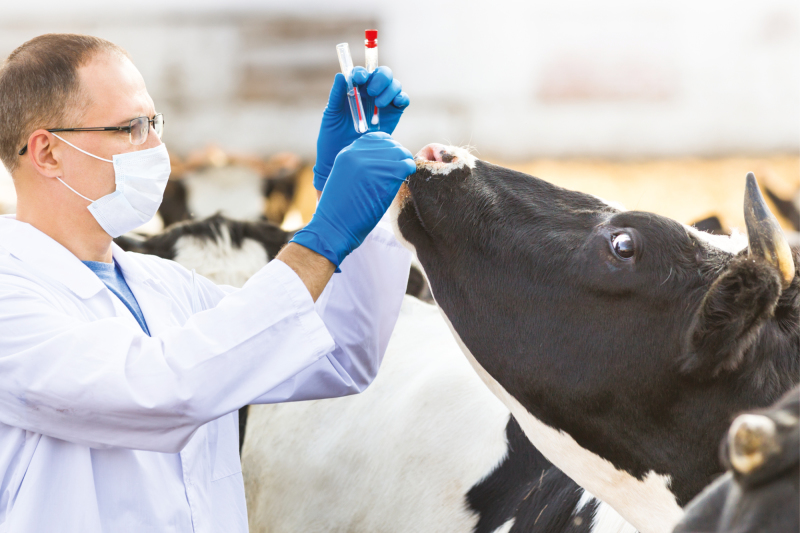
A veterinarian caring for a dairy cow.

Recently, care ethics has been developed as an ethical approach (Loewy and Springer, 2004). I agree with Hans Harbers (2009) that care ethics is the most promising integrative framework for ethics of animal production systems. Care ethics focuses on values that are important for the maintenance and flourishing of (care) relationships, such as commitment, dependency, responsibility, and care (Devettere, 2009). An important aim of caring is to create shared values for all stakeholders involved in the production chain. In agricultural systems, people care for plants and animals in the two senses of the word “care”: “care for” and “care about” (being concerned; Figure 3).

Good farming is a matter of endless care, in various shapes and sizes (Scholten et al., 2013). Good care requires the involvement of all stakeholders in the production chain, but also of citizens, consumers, civil society, and government (Harbers, 2009). As a consequence, care is always accompanied by societal concerns. Although care is firmly embedded in economic activity, this does not automatically imply the primacy of the economy (Harbers, 2009). In my view, caring also means the responsibility to take care of the situation in farming by contributing to innovative processes and thereby contributing to society. This entails clarity about responsibilities as an essential element for an excellent organization of a caring farming system.

In a number of innovation areas, such as genomics, synthetic biology, and animal welfare, ethicists are asked to help to solve moral problems in the early stages of innovation (e.g., Singer, 1986). Can ethicists help to solve moral problems in the early stages of innovation? In the past, ethics often seemed to lag behind technical progress, and, according to Grunwald (2010), as a response ethics joined the move towards “upstream engagement” in the field of Science and Technology Studies. As early as 1980, David Collingridge wrote a book on the social control of technology with the objective of avoiding the harmful social consequences of a new technology (Collingridge, 1980). This may be done by changing technology in its infancy by imposing on it all kinds of controls and restrictions. Two conditions are necessary to avoid the undesired consequences of a new technology: “It must be known that a technology has, or will have, harmful effects, and it must be possible to change the technology in some way to avoid the effects” (Collingridge, 1980). One or both of the conditions are often lacking, and attempts to control technology seldom succeed: the “dilemma of control”. The first horn of the dilemma is that the harmful social consequences of the fully developed technology cannot be predicted with sufficient confidence to justify the imposition of control. The second horn of the dilemma is that, by the time a technology is sufficiently well developed and diffused for its unwanted social consequences to become apparent, it is no longer easily controlled. Control may still be possible, but it will have become very difficult, expensive, and slow. What happens is that society and the rest of its technology gradually adapt to the new technology, so that, when it is fully developed, any major change in this new technology requires changes in many other technologies and social and economic institutions, making its control very disruptive and expensive (Collingridge, 1980, 19).

An important assumption of the Collingridge dilemma is the consequentialist/utilitarian perspective in ethics. The normative starting point of the dilemma is the need to avoid the harmful social consequences of a technology, but the message of the dilemma is that a consequentialist/utilitarian perspective is impossible. In the early phases of a new technology, ethical deliberations become speculative because we lack the required knowledge (Grunwald, 2010). In the later phases of a new technology, ethical deliberations often come too late, namely, when all of the relevant decisions have already been made, when it is too late to avoid harmful consequences of the technology. Collingridge’s own normative response to the dilemma is to maintain the “freedom to control technology”, because the essence of controlling technology is to retain “… the ability to change a technology, even when it is fully developed and diffused, so that any unwanted social consequences it may prove to have can be eliminated or meliorated” (Collingridge, 1980, 20/21). He suggests developing organizational structures and scientific tools to deal with the resistance to such control (Collingridge, 1980, 19). Experts, decision makers, and end-users all are entangled in controlling the new technology.

However, Collingridge did not foresee that some experts were going to use a version of his control dilemma as a normative tool in their attempts to exclude prospective users from the innovation process. Experts sometimes stress that they are willing to include users in the early stages of the new technology (Gremmen, 2006), when there is still a lot of room to take the voice of prospective users into account in the design of the product, but the experts can offer little concrete information that would allow prospective users to imagine how they could integrate the end-product in their everyday life. This version of the Collingridge dilemma depicts the end-users in the emergence of new technologies as the end-point of a linear process. However, the world of the users and the world of technological innovation are by no means separate entities that only merge when a final product is delivered to the users; they are already entangled from the start. Technology assessment, and, later, constructive technology assessment, recognized the importance of involving users in the innovation process to encourage integration of new technologies in users’ everyday lives (Rip et al., 1995). The case has been made that technologists need to study responses to science in order to learn from them and to discover missing propositions in their own reasoning (Locke, 2002). Every day-life concerns that inform people’s responses to emergent technologies may be at odds with scientific and technological standards but can and should be understood on their own terms. In this way, experts could benefit from the active involvement of prospective users (Veen, 2010).

It is difficult for ethicists to assist innovators, because most normative ethical theories have problems in dealing with the future. Not only do the results of an innovation trajectory have unknown consequences, but, more importantly, we do not know the results of innovation at the start of the innovation trajectory. This means that, in moral reasoning about innovations in the making, the relevant moral facts and the appropriate principles are more or less still unknown, as also the relevant moral consequences. For that reason, I describe the ethics of innovation in animal production systems as emergent. Examples of the main characteristics of an emergent ethics of animal production systems are moral lock-in, the slippery slope argument, instrumentalization, and commodification. Only by doing ethics in life sciences will the moral dilemmas emerge in the trajectory of responsible innovation.

There are different kinds of ethical arguments about controversial agricultural technologies. On the critical side, some people have objections to a particular technology as such. In the case of genetic modification, for example, this argument amounts to the claim that it is unnatural and therefore morally problematic (Haperen et al. 2012). Many critics might not be so much opposed to Genetic Modification technology as such, but more to its different applications (Rollin, 2006). From a consequentialist stance, this means that even people who do not have an objection in principle to the technology can still be critical of its use in agriculture in general and in food production in particular (Sandoe and Christiansen, 2008). Current applications of agricultural biotechnology have also been criticized from the viewpoint of justice, in particular with respect to the distribution of economic benefits from its use (Thompson, 2007). Some critics emphasize the risks and uncertainties with this new technology and argue either that there are risks to human health or the environment, or that there might be such risks, and that for this reason, some version of the precautionary principle should be applied (Gremmen, 2006). Ethics may clarify and test such arguments and explicate normative and epistemic assumptions. In livestock farming, genetic modification may contribute to all kinds of efficiency benefits but, at the same time, may be used to circumvent certain ethical problems (Hanssen and Gremmen, 2013). The following example on moral lock-in in the case of killing one-day old male chicks illustrates this (Bruinis et al., 2015; Gremmen et al., 2018).

## Moral Lock-in and the Killing of Day-old Chicks

**Figure 4.**
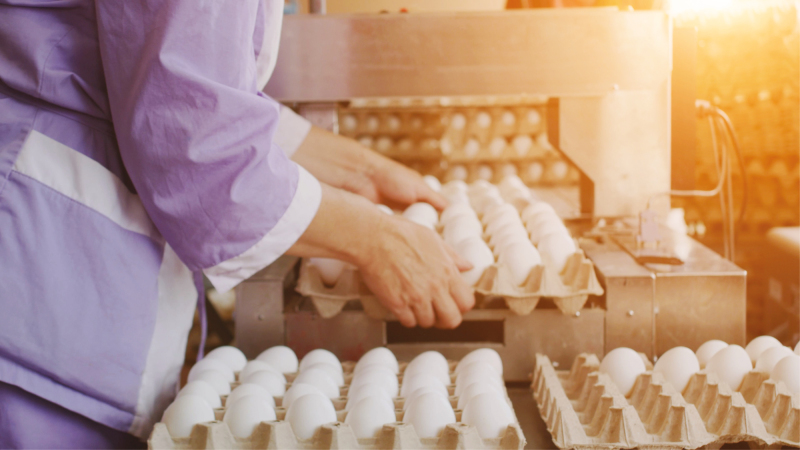
The process of sorting chicken eggs at a poultry farm.

In response to the increasing demand for safe and cheap food in sufficient quantities, the intensification and mechanization of poultry farming began in the mid-twentieth century. The number of chickens kept by any one farmer has increased considerably since then. Efficiency and specialization were enabled by developments in feeding, breeding, housing of the animals, and increased knowledge of veterinary medicine. Genetic selection enabled egg production by layer-type chickens and chicken meat production using specialized meat-type chickens. Therefore, male chicks from layer-type chickens became less attractive for meat production. With the available sexing techniques (Figure 4), which made it possible to distinguish males from females immediately after hatching, it became common practice to kill these male day-old chicks.

In the Netherlands, over 50 million male chickens are killed annually immediately after hatching. Societal opposition to this practice has prompted the development of innovations. Several alternatives to the killing of day-old chicks have been proposed (Leenstra et al., 2010); this leads to the question of whether these alternatives are morally superior. One alternative direction aims to use genetic modification in the breeding of laying hens in such a way that the hatching eggs containing males can easily be identified with spectroscopy, a non-invasive technique compared to the technique of taking a sample from the egg to find the difference between male and female eggs. The GM alternative takes advantage of the genetics of birds to ensure GM-free laying hens, and also that their eggs are GM free.

The killing of one-day-old male chicks is clear case of a morally inferior practice and has potentially morally better alternatives. Besides the GM alternative, there are several others: raising the male chicks, dual use of chickens, taking a sample from the egg, etcetera. Each alternative has its advantages and disadvantages with respect to technical and socio-ethical aspects, and each has a specific importance for various stakeholders. Solving one issue raised by the current situation throws up new issues. For example, by acknowledging arguments against the killing of such young animals and starting to rear the males, issues arise around the impact on the environment and the marketing of the chicks. The issue of killing day-old chicks and its alternatives thus seems to be an example of choosing the least of several possible evils and can be explained by a special type of moral lock-in.

Since the mid-1980s, technological lock-in has become an important subject of growing academic enquiry in the field of innovation studies, especially by economists working within an evolutionary tradition (David, 1985; Arthur, 1990). The general idea of lock-in is that technologies and technological systems follow specific paths that are difficult and costly to escape (Perkins, 2003). Even if potentially superior alternatives are available, these technologies and technological systems often survive for a very long time.

**Figure 5.**
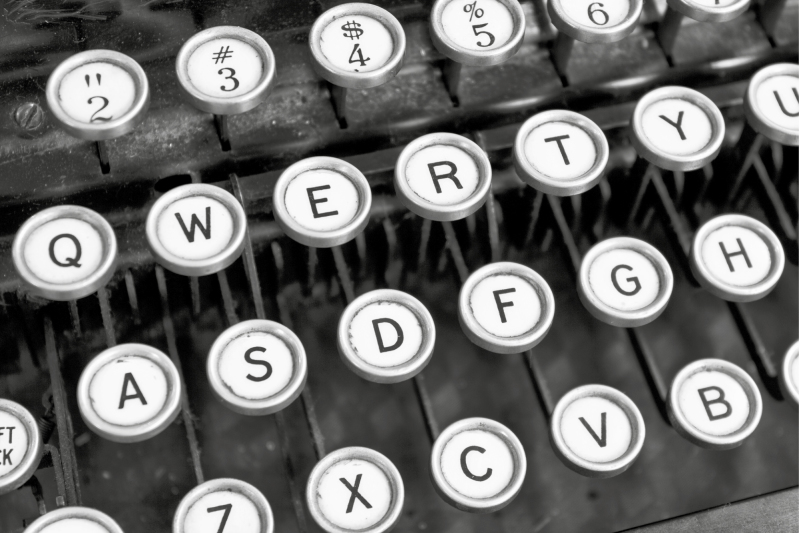
An antique typewriter with the traditional QWERTY key layout.

The famous examples in the literature are the triumph of the QWERTY keyboard layout (Figure 5) over the Dvorak Simplified Keyboard layout (David, 1985) and the race between VHS and Betamax as a video cassette recorder standard (Arthur, 1990). In the literature, lock-in is explained by the increasing returns of an initial lead in the competition between technologies (David, 1985; Arthur, 1989). “This arises because early adoption can generate a snowballing effect whereby the preferred technology benefits from greater improvement than its competitors, stimulating further adoption, improvement, and eventual leadership” (Perkins, 2003, 23).

There are many ways in which locked-in technologies may be inferior to their alternatives. We focus on *moral* lock-in: the way a production system can be locked-in to technology standards that are potentially morally inferior. In some cases, there is consensus on the potential for moral improvement that could be achieved through the development of alternative technologies. The question then becomes: What is holding back the development of these morally better technologies? Many debates about the transition to these new technologies focus only on the costs involved (Carrillo-Hermosilla, 2013). Our hypothesis is that a kind of moral lock-in may explain the survival of morally inferior technologies. We consider Responsible Innovation, a concept for balancing economic, sociocultural, and environmental aspects in innovation processes (EC, 2011), as an approach to morally “unlock” alternative innovations. By involving stakeholders in the innovation process and by considering ethical and societal aspects during this process, the socio-ethical acceptability and the societal desirability of innovative products will increase significantly (Blok and Lemmens, 2015).

## Conclusion

**Figure 6.**
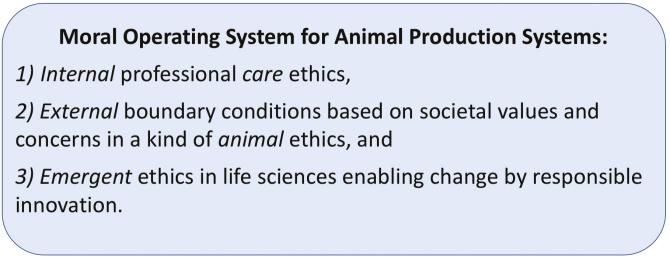
Moral operating system for animal production systems.

The upshot of this paper is that we need to develop a Moral Operating System of animal production systems (Figure 6). An ethics of livestock farming needs more than just animal ethics. We need an ethics of animal production “systems” consisting of three interactive, dynamic parts: an “internal” professional “care” ethics, “external” boundary conditions based on societal values and concerns in a kind of “animal” ethics, and an “emergent” ethics in life sciences enabling change by responsible innovation. Together, these three parts are the Moral Operating System of a production system. The aim is to adapt and broaden the existing ethical frameworks on agriculture to new scientific methods and technologies. This will help scientists, stakeholders, and policymakers to understand, evaluate, and monitor the integration of ethical aspects of agricultural systems.

## About the Author

Professor Dr **Bart Gremmen** is based at the Philosophy Chair Group, known for applying ethical theory to life science domains with a strong focus on plant, animal, and environmental ethics. His membership of the Plant Breeding group (2004-) and the Adaptation physiology group (2010-) and his collaboration with different Wageningen UR groups (environmental science; technology innovation; strategic communication) ensures a multidisciplinary approach. Professor Gremmen is a member of the environmental and ethics groups of the Netherlands Research School of Philosophy, and he has coordinated three large NWO genomics programs. He is member of the management team of the 4TU Centre for Ethics of Technology and has established a strong international network of environmental and applied ethicists. Professor Gremmen is president of the International Society for the Hermeneutics of science. He is also chair of the Ethics working group of the European Association of Animal Scientists.
